# Performance evaluation of the Xpert MTB/RIF assay according to its clinical application

**DOI:** 10.1186/s12879-014-0589-x

**Published:** 2014-11-14

**Authors:** Hee Jae Huh, Byeong-Ho Jeong, Kyeongman Jeon, Won-Jung Koh, Chang-Seok Ki, Nam Yong Lee

**Affiliations:** Department of Laboratory Medicine and Genetics, Sungkyunkwan University School of Medicine, 81 Irwon-ro, Gangnam-gu, Seoul Republic of Korea; Division of Pulmonary and Critical Care Medicine, Department of Medicine Samsung Medical Center, Sungkyunkwan University School of Medicine, 81 Irwon-ro, Gangnam-gu, Seoul 135-710 Republic of Korea

**Keywords:** Mycobacterium tuberculosis, Pulmonary tuberculosis, Xpert MTB/RIF assay, Korea

## Abstract

**Background:**

The Xpert MTB/RIF assay (Xpert assay; Cepheid, Sunnyvale, CA) is becoming the test of choice for the rapid diagnosis of tuberculosis and rifampin (RIF) resistance. The aim of this study was to evaluate the performance of the Xpert assay with respect to its clinical application at a tertiary care hospital in Korea, a country with an intermediate tuberculosis burden and high-resource.

**Methods:**

A total of 303 Xpert assay results from 109 smear-positive and 194 smear-negative respiratory specimens were retrospectively reviewed. Based on patients’ medical records, four categories of clinical applications of the Xpert assay were identified: (1) the diagnosis of pulmonary tuberculosis in patients with a high probability of pulmonary tuberculosis according to their clinical and radiological features; (2) the exclusion of tuberculosis in clinically indeterminate patients for pulmonary tuberculosis; (3) the differentiation of *Mycobacterium tuberculsosis* (MTB) from nontuberculous mycobacteria in a smear-positive specimen; and (4) the diagnosis of RIF resistance. Standard culture and drug susceptibility tests were used as reference methods.

**Results:**

The sensitivity of the Xpert assay for MTB detection in category 1 was 89.8% (95% confidence interval [CI], 78.5-95.8%), but 66.7% (95% CI, 12.5-98.2%) in category 2. The positive predictive values ranged from 33.3% (95% CI, 6.0-75.9%) in category 2 to 91.3% and 91.7% in categories 1 and 3, respectively. The negative predictive values were over 90% in all categories. The Xpert assay correctly detected RIF resistance in six of the seven (85.7%) isolates tested.

**Conclusions:**

The Xpert assay exhibited variable performance according to its clinical application; this finding cautions that careful interpretation for the results of this assay would be needed according to its intended purpose.

**Electronic supplementary material:**

The online version of this article (doi:10.1186/s12879-014-0589-x) contains supplementary material, which is available to authorized users.

## Background

The Xpert MTB/RIF assay (Xpert assay; Cepheid, Sunnyvale, CA) is a fully automated, cartridge-based, real-time polymerase chain reaction (PCR) assay designed to detect the presence of *Mycobacterium tuberculosis* (MTB) and rifampin (RIF) resistance within 2 hours [1],[2]. The Xpert assay can not only be utilized as a rapid diagnostic test for tuberculosis in patients with presumptive pulmonary tuberculosis, but also can be used to rapidly exclude tuberculosis (e.g., for determining airborne infection isolation discontinuation) or to differentiate MTB from nontuberculous mycobacteria (NTM) in smear-positive cases. In addition, this assay may be used to determine whether a patient with tuberculosis is infected with a RIF-resistant strain [3]–[6].

According to the 2013 World Health Organization Global Tuberculosis Report, South Korea is classified as a high-income country with an intermediate tuberculosis burden, including an incidence rate of 108 per 100,000 inhabitants in 2013 and 1,212 cases of confirmed multidrug-resistant tuberculosis reported in 2012 [7]. The accuracy of the Xpert assay and its effectiveness in the rapid diagnosis of tuberculosis have both been demonstrated in previous studies [3]–[5],[8]–[11]. However, the performance of the Xpert assay has not yet been assessed in an intermediate-incidence, high-resource setting with respect to its different applications [9]–[11].

In this study, we retrospectively evaluated the performance of the Xpert assay according to its clinical application in a tertiary care hospital in South Korea.

## Methods

### Study design

**Figure 1:**
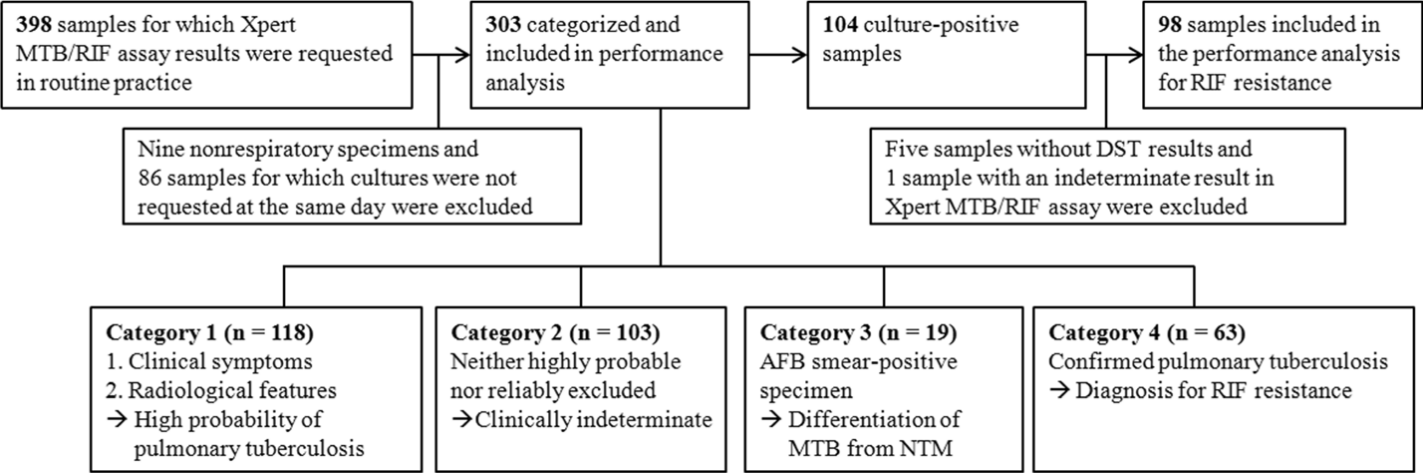
**Flow diagram outlining patient enrollment and stratification for the analysis of the diagnostic performance of the assay.** Abbreviations: DST, drug susceptibility testing; MTB, *Mycobacterium tuberculosis*; NTM, nontuberculous mycobacteria; RIF, rifampin.

This study was conducted at Samsung Medical Center, Seoul, South Korea, and the study protocol was approved by the Institutional Review Board (#2014-05-029). A total of 398 respiratory specimens were evaluated consecutively using the Xpert assay between Oct. 2012 and Feb. 2014, and the results were retrospectively analyzed. Samples from patients from whom mycobacterial cultures were not requested at the same day and nonrespiratory specimens were excluded from this study (Figure 1).

Patient medical records and microbiological test results, including acid-fast bacilli (AFB) smear, mycobacterial culture, and drug susceptibility testing (DST) were reviewed. Based on patient medical records, the samples were divided into four different categories based on the desired clinical application of the Xpert assay. Cases were categorized independently by two doctors. Disagreement in the interpretation of data required final consensus between both doctors. Category 1 consisted of samples from patients with a high probability of pulmonary tuberculosis, for whom rapid diagnosis of tuberculosis was required. Presumptive pulmonary tuberculosis was defined as the presence of the clinical symptoms (cough, fever, night sweats, or weight loss) and radiologic findings compatible with tuberculosis, in either a chest X-ray or a computed tomography scan. All patients with a high probability of pulmonary tuberculosis had both clinical and radiologic features highly indicative of tuberculosis; moreover, these features were unlikely to be caused by another disease. Category 2 consisted of samples from patients that the diagnosis of pulmonary tuberculosis was neither highly probable nor reliably excluded by clinicians. The patients in this clinically indeterminate group were asymptomatic or did not have radiologic features highly suggestive of tuberculosis. Category 3 consisted of samples from patients with an AFB smear-positive specimen, for which MTB needed to be differentiated from NTM. The Xpert assay was performed as add-on test in this category. Category 4 consisted of samples from patients with risk of multidrug-resistant tuberculosis such as previously treated tuberculosis or no improvement despite standard tuberculosis treatment.

### Xpert MTB/RIF Gassays

For the Xpert assay, either 1 mL of respiratory specimen (without decontamination) or 0.5 mL of respiratory specimen sediment prepared according to the N-acetyl-l-cysteine-NaOH (NALC-NaOH) method was used [12]. The Xpert assay was conducted according to the manufacturer’s instructions, as described previously [3].

### Mycobacterial staining and culture

Acid-fast staining was performed with an auramine-rhodamine fluorescent stain, followed by confirmation with Ziehl-Neelsen staining. Staining results were graded according to the US Centers for Disease Control recommendations. Specimens in which the AFB smear results were categorized as grades 1 to 4 were defined as smear-positive [13]. All patient specimens were cultured on two different types of media, solid and liquid, for 6 weeks. To this end, decontaminated samples were inoculated into a mycobacterial growth indicator tube (MGIT 960 system; Becton Dickinson, Sparks, MD) and also into 3% Ogawa agar (Shinyang, Seoul, Korea). All positive cultures were subjected to AFB smear to confirm the presence of AFB and to exclude contamination. In addition, positive liquid cultures were confirmed by both the presence of cord formation and by MPT64 antigen testing (SD BIOLINE TB Ag MPT64 Rapid; Standard Diagnostics Inc., Yongin-si, Gyeonggi-do, South Korea). If any of these tests yielded a negative result, an *rpoB*-specific PCR test using the MTB-ID V3 kit (YD Diagnostics, Yongin-si, Gyeonggi-do, South Korea) was performed to differentiate between MTB and NTM. Positive cultures found only on solid medium were also confirmed by conventional PCR testing.

### Detection of rifampin resistance

All MTB isolates were tested for resistance to RIF using the MGIT 960 system, and were also referred to the Korean Institute of Tuberculosis for conventional DST using the absolute concentration method with Löwenstein-Jensen medium [14],[15]. The critical concentrations for RIF resistance were 1.0 μg/mL and 40 μg/mL in the MGIT 960 system and the absolute concentration method, respectively. For all isolates yielding discrepant results, the *rpoB* gene was sequenced [16],[17].

### Statistical analysis

The sensitivity, specificity, positive predictive value (PPV), and negative predictive value (NPV) of the assay were calculated for each category. These parameters were based on the results from two reference methods, a concurrent culture test and DST using the absolute concentration method.

Statistical analyses were performed using SPSS software, version 21.0 (SPSS Inc., Chicago, IL) and the VassarStats website (http://vassarstats.net/).

## Results

After exclusion of nine nonrespiratory specimens and 86 samples for which cultures were not requested at the same day, a total of 303 respiratory specimens (264 sputum samples and 39 samples of bronchial washing or bronchoalveolar lavage fluid) from 300 patients were used to analyze the diagnostic performance of the assay. The median age of patients was 58 years (range, 18–93 years); 197 (65.7%) patients were male. Only one subject was infected with HIV.

A total of 109 (36.0%) of the 303 samples were smear-positive, while 194 were smear-negative, including 15 trace results and 179 negative results. Furthermore, 119 (39.3%) samples were positive for MTB according to the Xpert assay, whereas 104 samples (34.3%) gave positive culture results for MTB.

The overall performance of the Xpert assay and its performance according to sample smear status are shown in Table 1. When the 32 culture-negative samples from patients who were currently receiving tuberculosis treatment, in the 60 days prior to testing and started >48 hours ago, were excluded from analysis [3], the overall sensitivity, specificity, PPV, and NPV (95% confidence interval [CI]) of the Xpert assay were 91.3% (83.8-95.7%), 94.0% (89.0-96.9%), 90.5% (82.8-95.1%), and 94.6% (89.6-97.3%), respectively (Table 1). The sensitivity in smear-positive specimens was 96.2% (95% CI, 88.5-99.0%), but 76.0% (95% CI, 54.5-89.8%) in smear-negative specimens.

**Table 1:** **Performance of the Xpert MTB/RIF assay as stratified by smear status**

Performance of the Xpert MTB/RIF assay	Total ( *n* =303)	Total ( *n* =271) ^*a*^	Smear result (total, *n* =271) ^*a*^	
No./Total no.% (95% CI)			Smear-positive ( *n* =100)	Smear-negative ( *n* =171)
Sensitivity	95/104	95/104	76/79	19/25
	91.3 (83.8-95.7)	91.3 (83.8-95.7)	96.2 (88.5-99.0)	76.0 (54.5-89.8)
Specificity	175/199	157/167	18/21	139/146
	87.9 (82.4-92.0)	94.0 (89.0-96.9)	85.7 (62.6-96.2)	95.2 (90.0-97.9)
PPV	95/119	95/105	76/79	19/26
	79.8 (71.3-86.4)	90.5 (82.8-95.1)	96.2 (88.5-99.0)	73.1 (51.9-87.6)
NPV	175/184	157/166	18/21	139/145
	95.1 (90.6-97.6)	94.6 (89.6-97.3)	85.7 (62.6-96.2)	95.9 (90.8-98.3)

Abbreviations: CI, confidence interval; NPV, negative predictive value; PPV, positive predictive value.

^*a*^Excluding the 32 culture-negative samples from patients who were currently receiving anti-tuberculosis treatment.

The overall performance of the Xpert assay and its performance according to its clinical application are shown in Table 2. Variable performance of Xpert assay was observed between categories. The sensitivity of the Xpert assay for MTB detection in category 1 was 89.8% (95% CI, 78.5-95.8%) but was 66.7% (95% CI, 12.5-98.2%) in category 2. Only 3 samples in category 2 were culture-positive and 5 samples were smear-positive: 1 sample of grade 4, 1 sample of grade 2, and 3 samples of grade 1. The PPV of the assay ranged from 33.3% (95% CI, 6.0-75.9%) in category 2 to 91.3% and 91.7% in categories 1 and 3, respectively. The lower specificity and PPV of the assay when used for category 4 samples was likely due to the tuberculosis treatment that the patients were receiving (53/63, 84.1%). A total of 14 false positive samples were all from patients who were currently receiving tuberculosis treatment. Of these, 8 samples (57%) were smear-positive: 1 sample of grade 3, 4 samples of grade 2, and 3 samples of grade 1. NPVs were over 90% in all categories.

**Table 2:** **Performance of the Xpert MTB/RIF assay as stratified by clinical application**

Performance of the Xpert MTB/RIF assay	Total ( *n* =303)	Clinical application			
No./Total no.% (95% CI)		Category 1	Category 2	Category 3	Category 4
		( *n* =118)	( *n* =103)	( *n* =19)	( *n* =63)
Sensitivity	95/104	53/59	2/3	11/11	29/31
	91.3 (83.8-95.7)	89.8 (78.5-95.8 )	66.7 (12.5-98.2)	100 (67.9-100)	93.5 (77.2-98.9)
Specificity	175/199	54/59	96/100	7/8^*a*^	18/32
	87.9 (82.4-92.0)	91.4 (80.3-96.8)	96.0 (89.5-98.7)	87.5 (46.7-99.3)	56.3 (37.9-73.2)
PPV	95/119	53/58	2/6	11/12^*a*^	29/43
	79.8 (71.3-86.4)	91.4 (80.3-96.8)	33.3 (6.0-75.9)	91.7 (59.8-99.6)	67.4 (51.3-80.5)
NPV	175/184	54/60	96/97	7/7	18/20
	95.1 (90.6-97.6)	90.0 (78.8-95.9)	99.0 (93.6-99.9)	100 (56.1-100)	90.0 (66.9-98.2)

Abbreviations: CI, confidence interval; NPV, negative predictive value; PPV, positive predictive value.

^*a*^One false positive sample showed no growth in mycobacterial culture.

Drug susceptibility culture results were available for 98 of the 104 MTB culture-positive samples, with 7 isolates (7.1%) resistant to RIF. The Xpert assay correctly detected RIF resistance in 6 out of the 7 resistant samples. Thus, the sensitivity of the Xpert assay was 85.7%. The assay also yielded one false positive result for RIF resistance. Therefore, the specificity of the assay was 98.9% (Table 3). Of the two specimens exhibiting a discrepancy between the Xpert assay and the phenotypic DST results, one isolate was identified as RIF-resistant by the Xpert assay but was phenotypically susceptible. Sequencing of the *rpoB* gene from this isolate identified a mutation at locus 516 (Asp → Tyr). The other isolate, which was phenotypically resistant and did not have an *rpoB* gene mutation as assessed by the Xpert assay, was revealed by sequencing to be consistent with the wild-type strain.

**Table 3:** **Performance of the Xpert MTB/RIF assay in the detection of rifampin resistance**

Rifampin resistance	Performance of the Xpert MTB/RIF assay			
	Sensitivity	Specificity	PPV	NPV
Total (*n* =98)				
No./Total no.% (95% CI)	6/7	90/91	6/7	90/91
	85.7 (42.0-99.2)	98.9 (93.2-99.9)	85.7 (42.0-99.2)	98.9 (93.2-99.9)

Abbreviations: CI, confidence interval; NPV, negative predictive value; PPV, positive predictive value.

## Discussion

Recent studies have highlighted the need for evaluating the performance of the Xpert assay in different settings, such as areas with different incidences of tuberculosis and different levels of medical resource [18]–[20]. These evaluations are important for determining whether the Xpert assay is transferrable to different settings. Sohn *et al*. reported that the impact of Xpert assay in a low-incidence, high-resource ambulatory setting is limited [18]. The present study was performed in South Korea, a country with an intermediate tuberculosis burden and a high level of medical resources. The results presented here demonstrate that the Xpert assay does not perform equally well for all clinical applications of MTB detection. A previous study reported that the Xpert assay performed equally well among patients with and without presumptive tuberculosis in a country highly endemic for tuberculosis [8]. These authors suggested the use of the Xpert assay as routine tuberculosis screening. However, in the present study, significant differences in the performance of the Xpert assay were observed when it was used to test samples from patients with a high probability of pulmonary tuberculosis (category 1) and those in clinically indeterminate category 2. A low PPV was noted in the low prevalence population group (category 2). Of the six positive samples, four (66.7%) were false positives (Xpert assay positive and culture-negative); all of the semi-quantitative results given by the Xpert assay were “Very Low” (*n* =3) or “Low” (*n* =1). This suggests the limited potential impact of Xpert assay to detect MTB in patients with a low probability of pulmonary tuberculosis in an intermediate burden setting where (1) routine laboratory smear and culture procedures are performed according to the standard diagnostic algorithm; (2) nucleic acid amplification tests (NAATs) in the laboratory performs well; and (3) experienced physicians care for tuberculosis patients. However, the Xpert assay showed superior specificity and a better NPV for category 2 samples compared with category 1 samples. Therefore, the Xpert assay is a suitable tool for the exclusion of tuberculosis in patients with a low probability of pulmonary tuberculosis.

In our study, the sensitivity (91.3%) of the Xpert assay for the diagnosis of pulmonary tuberculosis was comparable to the pooled sensitivity (89%; 95% credible interval [CrI], 85-92%) in a recent meta-analysis [21]. As expected, a higher proportion of smear-negative results (6/9) in Xpert-negative and culture-positive specimens were noted in our study, consistent with what has been reported previously [22]. With regard to specificity, 10 specimens were Xpert-positive and culture-negative. Five of these specimens were positive by at least one other NAAT, or culture-positive using a follow-up culture specimen.

Phenotypic DST is the gold standard, and has hitherto not been questioned [23]. However, concerns have been raised that some phenotypic DST methods are limited in their detection of certain *rpoB* mutations that result in RIF resistance [24]–[26]. In this study, 7 isolates were identified as RIF-resistant by the Xpert assays; 6 of these isolates had confirmed resistance according to culture-based DST. Sequencing of the *rpoB* gene of the isolate with the discrepant result revealed a mutation (Asp516Tyr) that had recently been suggested to be associated with increased treatment failure or relapse rates [24]. This finding suggests that the use of phenotypic DST as the gold standard for RIF resistance should be reconsidered, in the light of our confirmation of the Xpert assay.

The frequency of isolation of NTM from clinical specimens has shown a continuous increase in Korea [27]–[30]. A high specificity value is indispensable in order to discriminate MTB from NTM in smear-positive samples especially in countries with a high percentage of NTM isolates [6]. Furthermore, rapid discrimination between MTB and NTM can significantly decrease airborne infection isolation time for individuals hospitalized without active tuberculosis [31],[32]. In this respect, our study revealed that Xpert assay in smear-positive samples well-discriminated MTB from NTM without cross-reactivity with NTM species, albeit the small sample number.

The present study did have some limitations. First, this was a retrospective study in routine clinical practice at a single institution. Majority of the AFB smear were performed only in specimen for culture. Nevertheless, the retrospective design allowed us to understand the real situation regarding the implementation of the Xpert assay in routine clinical laboratories. Second, the strength of our findings might be somewhat limited by the small numbers of positive results, particularly in categories 2 and 3. To strengthen our results, prospective studies in these patient groups would be needed. However, this study successfully provided a foundation for the design of more comprehensive studies to evaluate the performance of Xpert assay with respect to its clinical application.

## Conclusion

The retrospective study present here revealed that the Xpert assay exhibited variable performance according to its clinical application in an intermediate-incidence, high-resource setting. The finding in this study cautions that careful interpretation for the results of this assay would be needed in the light of the intended purpose of the test.

## Authors’ contributions

NYL, WJK and CSK conceived the concept and design of the study. Data was categorized by BHJ and KJ. HJH performed the data analysis. HJH, WJK and CSK wrote the manuscript. All authors read and approved the final manuscript.

## Authors’ original submitted files for images

Below are the links to the authors’ original submitted files for images. Authors’ original file for figure 1
