# Supplementary figures and images for: Performance evaluation of the Xpert MTB/RIF assay according to its clinical application
Source: BMC Infect Dis. 2014 Nov 14;14:589. doi: 10.1186/s12879-014-0589-x (PMC4247199; doi:10.1186/s12879-014-0589-x)

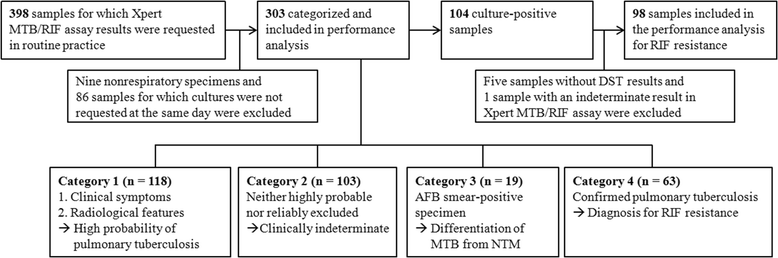

Supplement: Supplementary file 1 — Authors’ original file for figure 1 [file 12879_2014_589_MOESM1_ESM.gif]
